# Absolute configuration of (1*S*,2*S*)-3-methyl-2-phenyl-2,3-dihydro­thia­zolo[2,3-*b*]quinazolin-5-one

**DOI:** 10.1107/S160053681200832X

**Published:** 2012-03-03

**Authors:** Mostafa. M. Ghorab, Mansour. S. Al-Said, Maged. S. Abdel-Kader, Madhukar Hemamalini, Hoong-Kun Fun

**Affiliations:** a Medicinal, Aromatic and Poisonous Plants Research Center, College of Pharmacy, King Saud University, Riyadh 11451, Saudi Arabia; b Department of Pharmacognosy, College of Pharmacy, Salman Bin Abdulaziz University, PO Box 173, Al-Kharji 1194, Saudi Arabia; c X-ray Crystallography Unit, School of Physics, Universiti Sains Malaysia, 11800 USM, Penang, Malaysia

## Abstract

The absolute structure of the molecule in the crystal of the title compound, C_17_H_14_N_2_OS, was determined by the refinement of the Flack parameter to 0.0 (2) based on 1011 Friedel pairs. The quinazoline ring is essentially planar, with a maximum deviation of 0.037 (2) Å. The thia­zole ring is distorted from planarity [maximum deviation = 0.168 (2) Å] and adopts a slightly twisted envelope conformation, with the C atom as the flap atom. The central thia­zole ring makes dihedral angles of 7.01 (8) and 76.80 (10)° with the quinazoline and phenyl rings, respectively. The corresponding angle between the quinazoline and phenyl rings is 3.74 (9)°. In the crystal, there are no classical hydrogen bonds but stabilization is provided by weak C—H⋯π inter­actions, involving the centroids of the phenyl rings.

## Related literature

For details and applications of quinazoline derivatives, see: Ghorab *et al.* (2010*a* ▶,*b* ▶,*c* ▶). For related crystal structures, see: Al-Salahi *et al.* (2012 ▶); Priya *et al.* (2011 ▶); Liu *et al.* (2010 ▶). For ring conformations, see: Cremer & Pople (1975 ▶). For bond-length data, see: Allen *et al.* (1987 ▶).
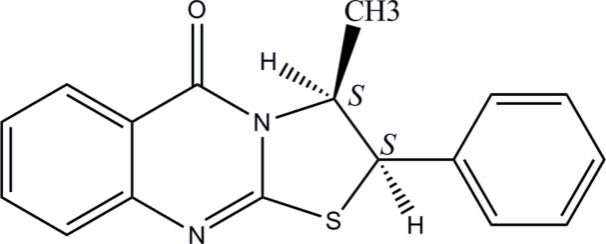


## Experimental

### 

#### Crystal data

C_17_H_14_N_2_OS
*M* *_r_* = 294.36
Orthorhombic, 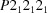

*a* = 8.4865 (1) Å
*b* = 10.0846 (2) Å
*c* = 16.8290 (3) Å
*V* = 1440.28 (4) Å^3^
*Z* = 4
Cu *K*α radiation
μ = 1.99 mm^−1^
*T* = 296 K
0.96 × 0.64 × 0.51 mm

#### Data collection

Bruker SMART APEXII CCD diffractometer
Absorption correction: multi-scan (*SADABS*; Bruker, 2009 ▶) *T*_min_ = 0.251, *T*_max_ = 0.431
8654 measured reflections
2637 independent reflections
2521 reflections with *I* > 2σ(*I*)
*R*_int_ = 0.028

#### Refinement

*R*[*F* ^2^ > 2σ(*F* ^2^)] = 0.043
*wR*(*F* ^2^) = 0.109
*S* = 1.08
2637 reflections
192 parameters
H-atom parameters constrained
Δρ_max_ = 0.22 e Å^−3^
Δρ_min_ = −0.23 e Å^−3^
Absolute structure: Flack (1983 ▶), with 1011 Friedel pairs
Flack parameter: 0.00 (2)

### 

Data collection: *APEX2* (Bruker, 2009 ▶); cell refinement: *SAINT* (Bruker, 2009 ▶); data reduction: *SAINT*; program(s) used to solve structure: *SHELXTL* (Sheldrick, 2008 ▶); program(s) used to refine structure: *SHELXTL*; molecular graphics: *SHELXTL*; software used to prepare material for publication: *SHELXTL* and *PLATON* (Spek, 2009 ▶).

## Supplementary Material

Crystal structure: contains datablock(s) global, I. DOI: 10.1107/S160053681200832X/nk2145sup1.cif

Structure factors: contains datablock(s) I. DOI: 10.1107/S160053681200832X/nk2145Isup2.hkl

Additional supplementary materials:  crystallographic information; 3D view; checkCIF report

## Figures and Tables

**Table 1:** Hydrogen-bond geometry (Å, °) *Cg*3 and *Cg*4 are the centroids of the C4–C9 and C11–C16 phenyl rings, respectively.

*D*—H⋯*A*	*D*—H	H⋯*A*	*D*⋯*A*	*D*—H⋯*A*
C2—H2*B*⋯*Cg*3^i^	0.98	2.91	3.824 (2)	155
C6—H6*A*⋯*Cg*4^ii^	0.93	2.81	3.637 (3)	146

Symmetry codes: (i) 
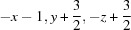
; (ii) 
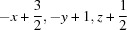
.

**Fig. 1.**
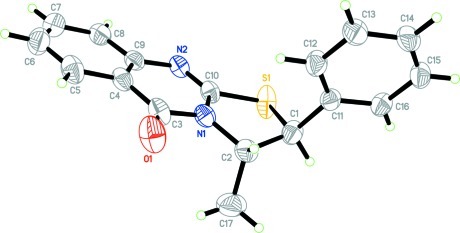
The asymmetric unit of the title compound, showing 30% probability displacement ellipsoids.

**Fig. 2.**
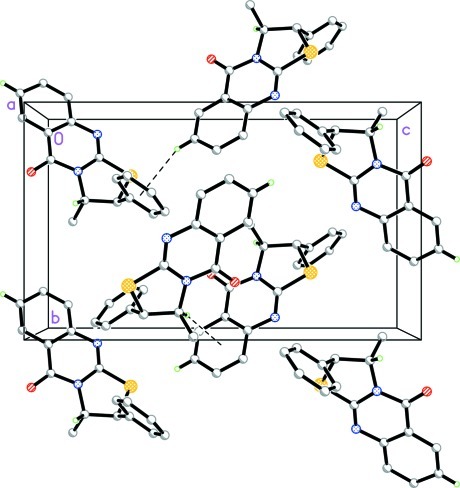
The crystal packing of the title compound (I).

## supplementary crystallographic information

### Comment

Quinazoline derivatives are well known as biologically active compounds. Consequently, quinazolines have been intensively studied for their interesting pharmacological properties such as anticancer activity (Ghorab *et al.*, 2010*a*,*b*,*c*). The crystal structures of 2-Ethoxy-5-methylbis[1,2,4]triazolo[1,5-*a*:4',3'-*c*] quinazoline (Al-Salahi *et al.*, 2012), 3-(4-Chlorophenyl)quinazolin-4(3*H*)-one (Priya *et al.*, 2011) and 2-Anilino-3-(2-hydroxyphenyl)quinazolin-4(3*H*)-one methanol monosolvate (Liu *et al.*, 2010) have been reported in the literature. Herein, we report the crystal structure of title compound (I).

The asymmetric unit of the title compound is shown in Fig. 1. The quinazoline (N1,N2/C3–C10) ring is essentially planar, with a maximum deviation of 0.037 (2) Å for atom C8. The thiazole (S1/N2/C7–C9) rings adopt an envelope conformation with the C2 (0.168 (2) Å) atom as the flap atom and with puckering parameter, Q = 0.2724 (19) Å and θ = 226.7 (4)° (Cremer & Pople, 1975). The central thiazole (S1/N1/C1–C2,C10) ring makes dihedral angles of 7.01 (8)° and 76.80 (10)° with the quinazoline (N1,N2/C3–C10) and phenyl (C11–C16) rings, respectively. The corresponding angle between the quinazoline (N1,N2/C3–C10) and phenyl (C11–C16) rings is 73.74 (9)°. The bond lengths (Allen *et al.*, 1987) and angles are within normal ranges.

The absolute configuration of the molecule were determined by the refinement of the Flack parameter to 0.0 (2). There are two chiral centres in the molecule. From the structure presented, these centers exhibit the following chiralities: C1 = S and C2 = S.

In the crystal structure (Fig. 2), there are no classical hydrogen bonds but stabilization is provided by weak C—H···π interactions (Table 1) involving the centroids of the (C4–C9) and (C11–C16) phenyl rings.

### Experimental

A mixture of 2-isothiocyanatobenzoate (1.93 g, 0.01 mole) and 2-amino-1-phenylpropan-1-ol (1.51 g, 0.01 mole) in dry dimethylformamide (30 ml) containing a catalytic amount of triethylamine was refluxed for 6 h. The solid obtained was recrystallized from ethanol to give the title thiazoloquinazline derivative compound. Single crystals suitable for X-ray structural analysis were obtained by slow evaporation from ethanol at room temperature.

### Refinement

All H atoms were positioned geometrically [C—H = 0.93–0.98 Å] and were refined using a riding model, with *U*_iso_(H) = 1.2 or 1.5*U*_eq_(C). A rotating group model was applied to the methyl groups. 1011 Friedel pairs were used to determine the absolute configuration.

### Crystal data

**Table:**

C_17_H_14_N_2_OS	*F*(000) = 616
*M**_r_* = 294.36	*D*_x_ = 1.358 Mg m^−^^3^
Orthorhombic, *P*2_1_2_1_2_1_	Cu *K*α radiation, λ = 1.54178 Å
Hall symbol: P 2ac 2ab	Cell parameters from 4079 reflections
*a* = 8.4865 (1) Å	θ = 4.4–71.7°
*b* = 10.0846 (2) Å	µ = 1.99 mm^−^^1^
*c* = 16.8290 (3) Å	*T* = 296 K
*V* = 1440.28 (4) Å^3^	Block, colourless
*Z* = 4	0.96 × 0.64 × 0.51 mm

### Data collection

**Table:**

Bruker SMART APEXII CCD diffractometer	2637 independent reflections
Radiation source: fine-focus sealed tube	2521 reflections with *I* > 2σ(*I*)
Graphite monochromator	*R*_int_ = 0.028
φ and ω scans	θ_max_ = 71.9°, θ_min_ = 5.1°
Absorption correction: multi-scan (*SADABS*; Bruker, 2009)	*h* = −10→10
*T*_min_ = 0.251, *T*_max_ = 0.431	*k* = −8→12
8654 measured reflections	*l* = −20→20

### Refinement

**Table:**

Refinement on *F*^2^	Hydrogen site location: inferred from neighbouring sites
Least-squares matrix: full	H-atom parameters constrained
*R*[*F*^2^ > 2σ(*F*^2^)] = 0.043	*w* = 1/[σ^2^(*F*_o_^2^) + (0.0729*P*)^2^ + 0.0628*P*] where *P* = (*F*_o_^2^ + 2*F*_c_^2^)/3
*wR*(*F*^2^) = 0.109	(Δ/σ)_max_ = 0.001
*S* = 1.08	Δρ_max_ = 0.22 e Å^−^^3^
2637 reflections	Δρ_min_ = −0.23 e Å^−^^3^
192 parameters	Extinction correction: *SHELXTL* (Sheldrick, 2008), Fc^*^=kFc[1+0.001xFc^2^λ^3^/sin(2θ)]^-1/4^
0 restraints	Extinction coefficient: 0.107 (4)
Primary atom site location: structure-invariant direct methods	Absolute structure: Flack (1983), with 1011 Friedel pairs
Secondary atom site location: difference Fourier map	Flack parameter: 0.00 (2)

### Special details

**Table:**

Geometry. All s.u.'s (except the s.u. in the dihedral angle between two l.s. planes) are estimated using the full covariance matrix. The cell s.u.'s are taken into account individually in the estimation of s.u.'s in distances, angles and torsion angles; correlations between s.u.'s in cell parameters are only used when they are defined by crystal symmetry. An approximate (isotropic) treatment of cell s.u.'s is used for estimating s.u.'s involving l.s. planes.
Refinement. Refinement of F^2^ against ALL reflections. The weighted R-factor wR and goodness of fit S are based on F^2^, conventional R-factors R are based on F, with F set to zero for negative F^2^. The threshold expression of F^2^ > 2σ(F^2^) is used only for calculating R-factors(gt) etc. and is not relevant to the choice of reflections for refinement. R-factors based on F^2^ are statistically about twice as large as those based on F, and R- factors based on ALL data will be even larger.

### Fractional atomic coordinates and isotropic or equivalent isotropic displacement parameters (Å^2^)

**Table:**

	*x*	*y*	*z*	*U*_iso_*/*U*_eq_	
S1	0.93740 (6)	0.77405 (8)	0.26159 (3)	0.0835 (3)	
N1	0.84292 (17)	0.75544 (18)	0.40678 (9)	0.0558 (4)	
N2	0.99073 (19)	0.57264 (18)	0.35894 (9)	0.0585 (4)	
O1	0.7394 (3)	0.7625 (3)	0.53134 (11)	0.0983 (7)	
C3	0.8142 (2)	0.7024 (3)	0.48182 (12)	0.0654 (5)	
C10	0.9264 (2)	0.6865 (2)	0.35080 (10)	0.0540 (4)	
C9	0.9715 (2)	0.51345 (19)	0.43270 (11)	0.0561 (4)	
C12	0.5242 (2)	0.8021 (2)	0.28662 (15)	0.0693 (6)	
H12A	0.5479	0.7496	0.3304	0.083*	
C11	0.6379 (2)	0.88563 (18)	0.25601 (12)	0.0556 (4)	
C4	0.8811 (2)	0.5710 (2)	0.49297 (11)	0.0598 (5)	
C13	0.3752 (3)	0.7954 (3)	0.25291 (17)	0.0743 (6)	
H13A	0.2993	0.7396	0.2746	0.089*	
C5	0.8607 (3)	0.5028 (3)	0.56509 (14)	0.0774 (6)	
H5A	0.8007	0.5405	0.6055	0.093*	
C14	0.3400 (3)	0.8704 (2)	0.18807 (17)	0.0730 (6)	
H14A	0.2396	0.8670	0.1660	0.088*	
C16	0.6023 (3)	0.9594 (2)	0.18898 (14)	0.0633 (5)	
H16A	0.6783	1.0144	0.1666	0.076*	
C6	0.9290 (4)	0.3807 (3)	0.57612 (17)	0.0875 (8)	
H6A	0.9131	0.3350	0.6235	0.105*	
C1	0.7973 (2)	0.9010 (2)	0.29489 (15)	0.0659 (5)	
H1A	0.8396	0.9878	0.2798	0.079*	
C15	0.4533 (3)	0.9515 (2)	0.15507 (14)	0.0723 (6)	
H15A	0.4299	1.0010	0.1100	0.087*	
C8	1.0433 (3)	0.3896 (2)	0.44566 (15)	0.0744 (6)	
H8A	1.1052	0.3517	0.4061	0.089*	
C2	0.7980 (2)	0.8926 (2)	0.38635 (14)	0.0659 (5)	
H2B	0.6911	0.9094	0.4060	0.079*	
C17	0.9107 (4)	0.9910 (3)	0.4252 (3)	0.1039 (11)	
H17A	0.9070	0.9806	0.4818	0.156*	
H17C	0.8802	1.0797	0.4113	0.156*	
H17D	1.0160	0.9746	0.4067	0.156*	
C7	1.0225 (4)	0.3247 (3)	0.51644 (19)	0.0874 (8)	
H7A	1.0707	0.2431	0.5248	0.105*	

### Atomic displacement parameters (Å^2^)

**Table:**

	*U*^11^	*U*^22^	*U*^33^	*U*^12^	*U*^13^	*U*^23^
S1	0.0541 (3)	0.1285 (5)	0.0680 (3)	0.0297 (3)	0.0168 (2)	0.0362 (3)
N1	0.0436 (7)	0.0702 (9)	0.0535 (8)	0.0037 (6)	−0.0037 (6)	−0.0028 (7)
N2	0.0529 (8)	0.0726 (10)	0.0500 (7)	0.0055 (7)	−0.0055 (6)	−0.0051 (7)
O1	0.1001 (14)	0.1344 (18)	0.0602 (9)	0.0463 (14)	0.0130 (8)	−0.0049 (10)
C3	0.0512 (9)	0.0952 (14)	0.0497 (9)	0.0092 (10)	−0.0022 (8)	−0.0043 (9)
C10	0.0393 (7)	0.0739 (11)	0.0490 (8)	0.0004 (7)	−0.0014 (7)	0.0020 (7)
C9	0.0526 (9)	0.0635 (10)	0.0522 (9)	−0.0071 (7)	−0.0092 (7)	−0.0034 (7)
C12	0.0519 (10)	0.0741 (12)	0.0820 (13)	−0.0033 (8)	−0.0106 (9)	0.0223 (10)
C11	0.0469 (8)	0.0523 (8)	0.0675 (10)	0.0055 (6)	−0.0032 (8)	0.0022 (8)
C4	0.0472 (8)	0.0815 (12)	0.0507 (9)	−0.0083 (8)	−0.0059 (7)	0.0028 (8)
C13	0.0491 (10)	0.0816 (13)	0.0923 (16)	−0.0055 (9)	−0.0084 (10)	0.0104 (12)
C5	0.0615 (12)	0.1126 (18)	0.0579 (11)	−0.0103 (12)	−0.0015 (9)	0.0107 (12)
C14	0.0527 (10)	0.0768 (13)	0.0896 (15)	0.0096 (9)	−0.0178 (11)	−0.0050 (11)
C16	0.0634 (11)	0.0560 (9)	0.0705 (11)	0.0068 (8)	−0.0005 (9)	0.0064 (8)
C6	0.0857 (16)	0.0934 (17)	0.0833 (15)	−0.0174 (14)	−0.0132 (14)	0.0307 (13)
C1	0.0460 (9)	0.0666 (10)	0.0850 (14)	−0.0021 (8)	−0.0040 (9)	0.0180 (10)
C15	0.0745 (13)	0.0697 (11)	0.0727 (12)	0.0158 (10)	−0.0151 (10)	0.0056 (9)
C8	0.0880 (15)	0.0662 (11)	0.0691 (11)	0.0015 (11)	−0.0152 (12)	−0.0065 (9)
C2	0.0505 (9)	0.0676 (11)	0.0795 (13)	0.0061 (8)	−0.0114 (9)	−0.0047 (10)
C17	0.0876 (19)	0.0806 (16)	0.143 (3)	−0.0012 (14)	−0.038 (2)	−0.0195 (18)
C7	0.107 (2)	0.0709 (13)	0.0845 (15)	−0.0052 (13)	−0.0216 (15)	0.0107 (11)

### Geometric parameters (Å, º)

**Table:**

S1—C10	1.7439 (18)	C5—C6	1.374 (5)
S1—C1	1.835 (2)	C5—H5A	0.9300
N1—C10	1.368 (3)	C14—C15	1.379 (4)
N1—C3	1.393 (3)	C14—H14A	0.9300
N1—C2	1.475 (3)	C16—C15	1.390 (3)
N2—C10	1.279 (3)	C16—H16A	0.9300
N2—C9	1.387 (3)	C6—C7	1.399 (5)
O1—C3	1.211 (3)	C6—H6A	0.9300
C3—C4	1.454 (3)	C1—C2	1.542 (3)
C9—C4	1.398 (3)	C1—H1A	0.9800
C9—C8	1.406 (3)	C15—H15A	0.9300
C12—C11	1.380 (3)	C8—C7	1.371 (4)
C12—C13	1.388 (3)	C8—H8A	0.9300
C12—H12A	0.9300	C2—C17	1.526 (3)
C11—C16	1.385 (3)	C2—H2B	0.9800
C11—C1	1.511 (3)	C17—H17A	0.9600
C4—C5	1.406 (3)	C17—H17C	0.9600
C13—C14	1.361 (4)	C17—H17D	0.9600
C13—H13A	0.9300	C7—H7A	0.9300
			
C10—S1—C1	93.18 (10)	C11—C16—H16A	119.9
C10—N1—C3	121.33 (18)	C15—C16—H16A	119.9
C10—N1—C2	116.71 (17)	C5—C6—C7	120.3 (2)
C3—N1—C2	121.72 (18)	C5—C6—H6A	119.9
C10—N2—C9	115.61 (16)	C7—C6—H6A	119.9
O1—C3—N1	121.6 (2)	C11—C1—C2	115.45 (17)
O1—C3—C4	124.9 (2)	C11—C1—S1	112.12 (16)
N1—C3—C4	113.49 (18)	C2—C1—S1	105.33 (14)
N2—C10—N1	127.10 (17)	C11—C1—H1A	107.9
N2—C10—S1	121.59 (14)	C2—C1—H1A	107.9
N1—C10—S1	111.31 (14)	S1—C1—H1A	107.9
N2—C9—C4	122.34 (19)	C14—C15—C16	120.2 (2)
N2—C9—C8	118.0 (2)	C14—C15—H15A	119.9
C4—C9—C8	119.6 (2)	C16—C15—H15A	119.9
C11—C12—C13	120.9 (2)	C7—C8—C9	120.2 (3)
C11—C12—H12A	119.5	C7—C8—H8A	119.9
C13—C12—H12A	119.5	C9—C8—H8A	119.9
C12—C11—C16	118.68 (18)	N1—C2—C17	110.36 (19)
C12—C11—C1	121.78 (18)	N1—C2—C1	106.58 (17)
C16—C11—C1	119.52 (18)	C17—C2—C1	113.2 (2)
C9—C4—C5	119.4 (2)	N1—C2—H2B	108.9
C9—C4—C3	119.94 (18)	C17—C2—H2B	108.9
C5—C4—C3	120.6 (2)	C1—C2—H2B	108.9
C14—C13—C12	120.0 (2)	C2—C17—H17A	109.5
C14—C13—H13A	120.0	C2—C17—H17C	109.5
C12—C13—H13A	120.0	H17A—C17—H17C	109.5
C6—C5—C4	120.2 (3)	C2—C17—H17D	109.5
C6—C5—H5A	119.9	H17A—C17—H17D	109.5
C4—C5—H5A	119.9	H17C—C17—H17D	109.5
C13—C14—C15	120.0 (2)	C8—C7—C6	120.3 (3)
C13—C14—H14A	120.0	C8—C7—H7A	119.8
C15—C14—H14A	120.0	C6—C7—H7A	119.8
C11—C16—C15	120.1 (2)		
			
C10—N1—C3—O1	−179.4 (2)	C3—C4—C5—C6	−177.7 (2)
C2—N1—C3—O1	6.4 (3)	C12—C13—C14—C15	1.1 (4)
C10—N1—C3—C4	0.1 (3)	C12—C11—C16—C15	1.8 (3)
C2—N1—C3—C4	−174.12 (17)	C1—C11—C16—C15	−176.6 (2)
C9—N2—C10—N1	0.6 (3)	C4—C5—C6—C7	1.6 (4)
C9—N2—C10—S1	−179.34 (13)	C12—C11—C1—C2	−34.8 (3)
C3—N1—C10—N2	−2.2 (3)	C16—C11—C1—C2	143.6 (2)
C2—N1—C10—N2	172.28 (18)	C12—C11—C1—S1	85.8 (2)
C3—N1—C10—S1	177.70 (15)	C16—C11—C1—S1	−95.81 (19)
C2—N1—C10—S1	−7.8 (2)	C10—S1—C1—C11	−105.81 (15)
C1—S1—C10—N2	171.57 (16)	C10—S1—C1—C2	20.52 (15)
C1—S1—C10—N1	−8.37 (15)	C13—C14—C15—C16	−1.6 (4)
C10—N2—C9—C4	3.2 (3)	C11—C16—C15—C14	0.1 (3)
C10—N2—C9—C8	−178.35 (18)	N2—C9—C8—C7	−177.4 (2)
C13—C12—C11—C16	−2.4 (4)	C4—C9—C8—C7	1.2 (3)
C13—C12—C11—C1	176.0 (2)	C10—N1—C2—C17	−99.9 (3)
N2—C9—C4—C5	177.15 (19)	C3—N1—C2—C17	74.6 (3)
C8—C9—C4—C5	−1.3 (3)	C10—N1—C2—C1	23.4 (2)
N2—C9—C4—C3	−5.2 (3)	C3—N1—C2—C1	−162.12 (17)
C8—C9—C4—C3	176.33 (19)	C11—C1—C2—N1	97.6 (2)
O1—C3—C4—C9	−177.2 (2)	S1—C1—C2—N1	−26.69 (19)
N1—C3—C4—C9	3.3 (3)	C11—C1—C2—C17	−140.9 (2)
O1—C3—C4—C5	0.4 (4)	S1—C1—C2—C17	94.8 (2)
N1—C3—C4—C5	−179.06 (19)	C9—C8—C7—C6	0.3 (4)
C11—C12—C13—C14	0.9 (4)	C5—C6—C7—C8	−1.7 (4)
C9—C4—C5—C6	0.0 (3)		

### Hydrogen-bond geometry (Å, º)

Cg3 and Cg4 are the centroids of the C4–C9 and C11–C16 phenyl rings, respectively.

**Table:**

*D*—H···*A*	*D*—H	H···*A*	*D*···*A*	*D*—H···*A*
C2—H2*B*···*Cg*3^i^	0.98	2.91	3.824 (2)	155
C6—H6*A*···*Cg*4^ii^	0.93	2.81	3.637 (3)	146

Symmetry codes: (i) −*x*−1, *y*+3/2, −*z*+3/2; (ii) −*x*+3/2, −*y*+1, *z*+1/2.
